# Dr. Felix's Remarkable Conversion of Diseases

**Published:** 1817-08

**Authors:** Mathias Felix


*Copy of a Letter from* Dr. FELIX, Royal Navy, *to* Dr. DICKSON, F. R. S. Ed. and L. S. Physician to the Fleet, &c. &c. &c.

DEAR SIR,

I Mentioned to you the other day, that an incident or two had occurred to me, in the early part of my practice, which, if not singular in their nature, are, at least, out of the ordinary routine.

Mr. Gottoch, a country gentleman, 70 years of age, of a full, corpulent habit, but enjoying general good health, excepting annual fits of the gout, which had regularly recurred for several years, and which he jocosely called his Doctor, was laid up with a paroxysm of the abovementioned disease, at Christmas 1787. Having occasion to pass through his domain, I made a friendly call, and found the old gentleman comfortably flannelled and bolstered up in bed; he was in high spirits, and said he had never before had *so agreeable a fit*, and insisted on my seeing his *rosy feet*. I remonstrated with him on the danger of exposure, as the weather was cold, with a severe frost on the ground; but he would have his way, and the feet were exhibited in a state of *gouty perfection*. The inspection was not long, and I soon afterwards quitted him, promising to return to dinner. I was back within the hour, and went up stairs to chat with my old friend, till dinner should be announced. He was still sitting up in bed, and we resumed our conversation; but I now observed him put his hand once or twice to his left side, and I at length asked him if he felt any uneasiness there. He said he believed he was a little tired, and would lie down. His servant was accordingly called to assist him; but he had scarcely lain down, when he was seized with so pungent a pain in the side, that he was obliged instantly to get up again. This did not afford relief; the pain increased; his breathing became difficult; and, in short, in a few minutes, there was every appearance of the most acute pleuritis! The face became flushed; the pulse rose strong. On turning up the bed-clothes, and removing the flannels, I found, to my astonishment, the swelling totally subsided; the redness gone; and the old gentleman's feet perfectly free from pain! The dinner had just been taken up, and as it was a family Christmas fête, plenty reigned in all parts. As every copper, boiler, and pot, had been put in requisition, there was no want of warm liquids; and therefore a large washing tub was ordered up, into which I had poured decoctions of ham, beef, &c. without any regard to the quality but the temperature of the ingredients. Into this heterogeneous mixture the old gentleman was immersed as high as the hips, and while there, I opened a vein and suffered him to bleed till he could breathe with freedom. By these prompt means I think his life was saved; but the case proved troublesome and tedious; the arthritic affection of the extremities was very partially and slowly brought back, and his *regular* attacks of gout were for ever afterwards suspended.

This retrocession or metastasis of arthritic matter or action was certainly very sudden, and strongly marked. From the time that his feet were shewn to me till my being obliged to take blood from the arm, two hours had not quite elapsed. Perhaps, however, the instance is not very singular or infrequent; but it is a necessary prelude to what follows. In short, it was the first link in a train of curious and anomalous symptoms which well deserve the attention of the pathologist and the practical physician.

The recovery of Mr. G. was slow, and his convalescence lengthened through the winter; but, as the summer advanced, he gradually recruited, and, to a certain degree, regained his usual health and spirits. His *regular* DOCTOR, however, returned no more, or paid him but hasty and unsatisfactory visits. About the close of the year 1788, he requested me to visit him, in order to consult me on a subject that had, for some time, given him much uneasiness, but which he had hitherto concealed. After much circumlocution, he unfolded this secret mischief, which turned out to be a well-marked hydrocele. After various consultations and explanations, he consented only to the palliative operation, which was performed, and full a quart of water was drawn off. As may be supposed, the serous collection returned, and the patient was urged to allow the radical cure to be effected; but he begged to have the temporary operation once more performed, promising that when the scrotum a third time filled, he would permit the injection to be employed for a permanent cure.

When the tumour had a third time attained nearly its usual maximum size, I examined it particularly, late one evening, and appointed the next morning for the operation. On arriving, I found him in bed, and having spread the necessary apparatus on a table, I removed the bedclothes, and exposed the scrotum; when, to my unspeakable surprize, as well as to the utter astonishment of my patient, not a vestige of the hydrocele remained! The scrotum was corrugated to its natural healthy size. Nothing particular had occurred during the night, which he passed in sound sleep, to account for the sudden disappearance of the tumour. He had voided no more urine than usual, nor had he had any evacuation from the bowels during the preceding twenty four hours. However this might be, my patient expressed himself right glad that Nature had saved me the trouble, and him the pain of the intended operation.

No perceptible effects immediately followed this extraordinary absorption; but after a few weeks, marks of general constitutional disorder began to manifest themselves. He became dyspeptic; complained of wandering pains, a short cough, and some difficulty in breathing, on walking up a hill, or going up stairs. His arms were particularly affected with pain; and, by degrees, he lost almost entirely the power of using them. At this time there was no swelling of the feet or ankles; but he constantly observed, that the greater the pain in his arms, the more free was the chest. It would be tiresome to narrate the various remedial measures pursued during a long period of suffering; suffice it to say, that the affection of the chest finally swallowed up all other complaints, and HYDROTHORAX became strongly and unequivocally developed. For a long time, medicine kept this distressing and dangerous disease in check; but at length, the constitution appeared to sink beneath its pressure, and his family were prepared for his decease: an event he himself fully expected; and for which he had made due preparation, by arranging all his temporal and spiritual concerns.

One night, when the last scene appeared to be closing,

I was requested by the family to wait till the mournful event was over, with which solicitation I complied, as every phenomenon indicated that dissolution was at hand. Through the day, the patient had been entirely deprived of speech. I sat up with him till late, and then prevailed on the family to retire to rest, promising to have them called when the awful moment of our friend's departure arrived. Finding that he still held out, I lay down about two o'clock in the morning, in an adjoining room, desiring the nurse to call me if any change took place. About seven she awoke me, to say that the patient was dying. I hastily repaired to the sick man's chamber, where I found the whole family assembled round his bed to witness the last scene. Placing myself at the bedside, I took his arm; but, instead of a pulse, an indistinct flutter only was occasionally perceptible. His eyes were fixed, and inanimate; a dewy sweat stood on his forehead; his breathing was laboured, and at long intervals; in short, he exhibited a perfect picture of a person in *articulo mortis*. In this state he continued upwards of half an hour, during which I kept my finger on his wrist, expecting the final stop, and thinking every attempt at respiration would be the last. During this distressing scene, he, to my astonishment—I may truly say *alarm*, opened his eyes, and starting suddenly up in the bed, fixed them, for several seconds, with fierceness, on one of his daughters, who sat petrified with fear near the bedside, and then sprang violently out, as if with intent to seize her, without my having the power, or the thought of preventing him! He fell all along, however, and then we recovered sufficient presence of mind to hurry to his assistance, and re-convey him to his bed. From this moment his breathing became free; his pulse returned; his speech was restored, but he was completely *deranged*, and continued so till his death, which did not take place till *fourteen months* after this eventful period!

During this long state of hallucination, I had constant opportunities of observing this unfortunate patient. The mental derangement varied much in character. At the commencement, it seemed more like delirium than mania; as it advanced, it often betrayed traits of whimsicality, often of fury. The first hallucination was an idea that he was at a particular inn, in a distant part of the country; and this impression he retained for some time, talking incessantly night and day: yet he exhibited no symptoms of pyrexia, or increased vascular action of any kind. After some months, he was attacked with *prurigo*, which proved very troublesome; but, during its continuance, there was an evident improvement in his general health. When this cutaneous eruption disappeared, anasarca supervened, and at length he died of general dropsy.

I leave you, Sir, to make your own comments on this remarkable series of conversions, assuring you only of the fidelity of the statement, and authenticity of the facts. I have seldom related the case, because, in general, it was listened to with an air of scepticism when detailed; but I cannot help thinking that it is not unworthy of record, or devoid of interest, both in a pathological and therapeutic point of view. I am, &c.

MATHIAS FELIX, M. D.

To Dr. Dickson, &c.

